# C-Peptide Promotes Cell Migration by Controlling Matrix Metallopeptidase-9 Activity Through Direct Regulation of β-Catenin in Human Endometrial Stromal Cells

**DOI:** 10.3389/fcell.2022.800181

**Published:** 2022-01-21

**Authors:** Sana Abdul Khaliq, Zobia Umair, Mi-Ock Baek, Seung Joo Chon, Mee-Sup Yoon

**Affiliations:** ^1^ Department of Molecular Medicine, Gachon University College of Medicine, Incheon, South Korea; ^2^ Department of Health Sciences and Technology, GAIHST, Gachon University, Incheon, South Korea; ^3^ Department of Obstetrics and Gynecology, Gachon University Gil Medical Center, College of Medicine, Gachon University, Incheon, South Korea; ^4^ Lee Gil Ya Cancer and Diabetes Institute, Gachon University, Incheon, South Korea

**Keywords:** C-peptide, migration, Akt, protein phosphatase 1, β-catenin, matrix metallopeptidase-9, metallopeptidase inhibitor 1

## Abstract

The motility of endometrial stromal cells (ESCs) contributes to the restoration of the endometrial functional layer and subsequently supports the trophoblast invasion during early pregnancy. Following ESCs differentiation through decidualization in response to progesterone during the menstrual cycle and embryo implantation, decidualized ESCs (D-ESCs) have greater motility and invasive activity. The human proinsulin-connecting peptide (C-peptide) is produced in equimolar amounts during the proteolysis of insulin in pancreatic β-cells. However, the function of C-peptide in the cellular motility of the human endometrium remains unexamined. In the present study, C-peptide was identified as a determinant of undecidualized human endometrial stromal cells (UnD-ESCs) migration. C-peptide promoted the migration and invasion of UnD-ESCs and trophoblast-derived Jeg3 cells, but not that of ESCs post decidualization, a functional and biochemical differentiation of UnD-ESCs. Both Akt and protein phosphatase 1 regulated β-catenin phosphorylation in UnD-ESCs, not D-ESCs, thereby promoting β-catenin nuclear translocation in C-peptide-treated UnD-ESCs. C-peptide was also observed to increase matrix metallopeptidase-9 (MMP9) activity by increasing MMP9 expression and decreasing the expression of metallopeptidase inhibitor 1 (TIMP1) and TIMP3. Their expression was modulated by the direct binding of β-catenin in the regulatory region of the promoter of MMP9, TIMP1, and TIMP3. Inhibition of either β-catenin or MMP9 dampened C-peptide-enhanced migration in UnD-ESCs. Together, these findings suggest that C-peptide levels are critical for the regulation of UnD-ESC migration, providing evidence for the association between C-peptide levels and the failure rate of trophoblast invasion by inducing abnormal migration in UnD-ESCs in hyperinsulinemia or PCOS patients.

## Introduction

The motility and invasion of endometrial stromal cells (ESCs) are essential for successful decidualization and human embryonic trophoblast invasion (Weimar et al., 2013; Vinketova et al., 2016), leading to the occurrence and maintenance of pregnancy (Gellersen et al., 2010). Decidualization is the differentiation of endometrial stromal cells (ESCs) in response to progesterone during the menstrual cycle and embryo implantation (Zhu et al., 2014). Decidualized ESCs (D-ESCs) undergo changes in transcription and morphology, preparing for prospective embryo implantation (Gellersen and Brosens, 2014). The invasive and migratory capacities of ESCs are essential for endometrial tissue remodeling during decidualization and implantation (Weimar et al., 2013), supported by the fact that decidualized ESCs exhibit greater invasive activity than undecidualized ESCs (UnD-ESCs) (Gellersen et al., 2010). Thus, successful implantation is associated with the well-regulated migration of D-ESCs, subsequent secretion of matrix metalloproteinases (MMPs) from D-ESCs, and the abundance of extra villous trophoblast cells (Grewal et al., 2008; Weimar et al., 2013; Macklon and Brosens, 2014). In addition, tissue inhibitors of MMPs (TIMP, also metallopeptidase inhibitor), a key regulator of MMP9 activity, play a key role in maintaining the homeostasis of endometrial migration and early implantation by altering trophoblast invasion (Fluhr et al., 2008).

The human proinsulin-connecting peptide (C-peptide) is produced by pancreatic β-cells through the proteolytic processing of proinsulin into insulin (Bhatt et al., 2014). C-peptide is composed of 31 amino acids in humans and is required for the proper folding of insulin and for the formation of interchain disulfide bonds in insulin, which stabilizes the A and B chains of the molecule (Wahren et al., 2000). C-peptide and insulin are secreted in equal amounts, and C-peptide is considered an inert biomarker for insulin level, based on the longer half-life of C-peptide than that of insulin (Hills and Brunskill, 2009). However, C-peptide has been reported to play a protective role in complications associated with diabetes (Bhatt et al., 2014); the multidimensional effects of C-peptide in animals and patients with diabetes are associated with multiple cell signaling pathways, including extracellular signal-regulated kinase 1/2 (ERK1/2) and Akt (Bhatt et al., 2014). Recently, we reported C-peptide as a negative determinant of decidualization (Khaliq et al., 2020), implying the role of C-peptide in the human endometrium. Our report also supports the association of high C-peptide levels in patients with polycystic ovarian syndrome (PCOS) with relatively inefficient decidualization (Chakraborty et al., 2013). High levels of C-peptide in the blood increase the risk of endometrial cancer, which is characterized by high potency of cell migration and invasion (Lukanova et al., 2004; Cust et al., 2007). C-peptide also promotes the migration of other cell types, such as endothelial cells and NIH3T3 cells (Lim et al., 2015). However, it remains unclear whether C-peptide affects ESC motility and regulates differentially in a differentiation-dependent manner.

Hence, we assess the motility of either UnD-ESCs or D-ESCs in the presence of C-peptide and explored the molecular mechanism underlaying the effects of C-peptide on cellular migration by comparing potential signaling characteristics. Our findings provide evidence that the C-peptide-induced molecular regulation may be associated with the abnormal migration of UnD-ESCs, and insights into the development of diagnostic or therapeutic strategies for infertility regarding cellular migration and invasion in the endometrium.

## Methods

### Antibodies and Other Reagents

C-peptide was obtained from Peptron (Daejeon, Republic of Korea). The following reagents were used in this study: Cell tracking dye-kit-Green-Cytopainter (ab138891) from Abcam (Cambridge, United Kingdom); Vectashield Antifade Mounting Medium with 4,6-diamidino-2-phenylindole (DAPI) (H1200) from VECTOR (Malven, PA, United States); okadaic acid (OA) (ALX-350-003-C100) from Cell Signaling Technology (Danvers, MA, United States); 8-bromo adenosine 3ʹ5ʹ-cyclic adenosine monophosphate (8-Br-cAMP) (B5386) and Akti (124018) from Sigma-Aldrich (St. Louis, MO, United States); PNU-74654 (74654) from Selleckchem (Houston, Texas, United States); and MMP2/MMP9 inhibitor (ab1415190) from Abcam. Antibodies against Akt (9272), pS473-Akt (4051), β-catenin (9582), pS522-β-catenin (9566), pS33/37/T41-β-catenin (9561), pT202/Y204-ERK1/2 (9101), ERK1/2 (9102), MMP2 (87809), MMP9 (13667), and PP2A (2259) were obtained from Cell Signaling Technology. Antibodies against tubulin (ab11304) and PP1A (MAB3000) were purchased from Abcam and R&D Systems (Minneapolis, MN, United States), respectively. Anti-mouse secondary antibody (115-035-003) and anti-rabbit antibody (211-002-171) were obtained from Jackson ImmunoResearch Laboratories Inc. (West Grove, PA, United States).

### Isolation of Human Endometrial Stromal Cells and Cell Culture

Human endometrial stromal cells (ESCs) were isolated from the endometria of 18 premenopausal women, aged 45–50 years, *via* hysterectomy. The participants exhibited no signs of glucose metabolism irregularities, diabetes, or PCOS, and underwent surgery for non-endometrial abnormalities at the Gil Hospital (Incheon, South Korea). All experiments were performed in accordance with the guidelines and regulations of Gachon University (GAIRB 2018-301), and all participants provided written informed consent. ESCs were isolated as previously reported (Yoon et al., 2007). The isolated cells were grown in a growth medium [Dulbecco’s modified Eagle’s medium (DMEM) containing 1 g/L glucose (Welgene, Gyeongsangbuk-do, Korea), 10% fetal bovine serum (FBS, Welgene), and 1% penicillin/streptomycin (P/S, 10,000 U/ml, Welgene)] at 37°C and 5% CO_2_, and detached from their plates using 0.05% trypsin-ethylenediaminetetraacetic acid (EDTA) (Welgene). *In vitro* decidualization was achieved by plating and expanding the cells to 100% confluency, followed by exposure to the differentiation medium (DMEM containing 1 g/L glucose, 10% FBS, 1% P/S, and 0.5 mM 8-Br-cAMP), which was replaced on alternate days. For C-peptide treatment, the cells were serum-starved for 48 h with serum-free DMEM overnight and treated with 50 nM C-peptide, as indicated in Figure Legends. Human placenta-derived Jeg-3 cells (30036) were obtained from the Korean Cell Line Bank and cultured in the growth medium of ESCs.

### Cell Lysis, Immunoprecipitation, and Western Blot Analysis

For western blot analysis, human endometrial stromal cells were washed with phosphate-buffered saline (PBS), lysed using a lysis buffer (9303, Cell Signaling Technology), and then microcentrifuged at 13,200 × *g* for 10 min at 4°C. The supernatant was collected and boiled in sodium dodecyl sulfate (SDS) sample buffer for 3 min. For the immunoprecipitation of β-catenin, cells were lysed in RIPA buffer I (50 mM Tris-HCl, pH 7.4, 150 mM NaCl, 0.5% sodium deoxycholate, 0.1% SDS, 1% NP-40, protease inhibitor cocktail (Cat. No. P8340, Sigma). The lysates were immunoprecipitated at 4°C with anti-β-catenin overnight, and then incubated with protein G-agarose (16-266, Millipore, Billerica, MA, United States) at 4°C for 1 h. The resulting beads were washed three times with RIPA buffer I. Proteins were resolved using SDS-polyacrylamide gel electrophoresis. The resolved gels were transferred to polyvinylidene fluoride membranes (Millipore). Each membrane was blocked with 5% (w/v) skim milk (232100, BD Life Sciences, Franklin Lakes, NJ, United States) in Tris-buffered saline (TBS) with 1% Tween for 30 min at room temperature (20–25°C) and then incubated overnight at 4°C with primary antibodies according to the manufacturer’s instructions. Immobilon Western Chemiluminescent HRP Substrate (Millipore) was used to detect horseradish peroxidase-conjugated secondary antibodies. The band intensities of the western blots were quantified using densitometry of X-ray film images using ImageJ (Khaliq et al., 2020).

### RNA Isolation and Quantitative Real Time Polymerase Chain Reaction

Total RNA was extracted from isolated HESCs using TRIzol reagent (Thermo Fisher Scientific, Waltham, MA, United States), and 25 ng/μl RNA was used to synthesize cDNA using TOPscript RT DryMIX (dT18 plus), according to the manufacturer’s instructions (Enzynomics, Daejeon, South Korea). Next, qRT-PCR was performed using TOPreal qPCR 2X PreMIX (SYBR Green with high ROX, Enzynomics) and a CFX384 C1000 thermal cycler (Bio-Rad, Hercules, CA, United States). Gene expression levels were normalized to human GAPDH expression levels.

The human primer sequences used in this study are shown in Supplementary Table S1.

### *In Vitro* Wound-Healing Scratch Assay

UnD-ESCs, D-ESCs, and Jeg-3 cells were incubated in 6 well plates of the growth medium until reaching 90–100% confluency. D-ESCs were used after incubation of UnD-ESCs with differentiation medium for 2 days. The cells were serum-starved by replacing the medium with serum-free DMEM for 18 h to minimize cell proliferation. Artificial wounds were established by scratching with clear edges across the wells using a sterile plastic pipette tip (200 µl). The cells were treated with or without 50 nM C-peptide for 24 h and stained using the CytoPainter Cell Tracking Staining Kit, according to the manufacturer’s instructions (Cho et al., 2019) and visualized using a fluorescent microscope with a ×4 objective (Olympus CKX3-Houn Microscope, Olympus, Tokyo, Japan). ImageJ was used to measure the number of migrated cells and the migration distances.

### Trans-Well and Invasion Assays

For trans-well assays, UnD-ESCs or D-ESCs were suspended in serum-free medium (DMEM that contained 1.0 g/L glucose and 1% P/S in a 24-well cell culture insert (pore size, 8 µm). Briefly, 300 μl of cell suspension (5 × 10^4^ cells) was added to the upper chamber, and 500 μl C-peptide-containing growth medium (DMEM with 1 g/L glucose, 10% FBS, 1% P/S, and 50 nM C-peptide) was added to the lower chamber. After 24 h of incubation, cells on the upper surface of the chamber were removed by wiping with a cotton swab. The migrated cells were fixed in 10% formaldehyde for 10 min and stained with DAPI. Images were captured using a fluorescent microscope (Olympus CKX3-Houn, 20X), and ImageJ was used to count the migrated cells in five randomly selected fields. Cell invasion was assessed using the QCM Collagen Cell Invasion Assay kit (ECM551, Millipore, Bedford, MA, United States), according to the manufacturer’s instructions. Briefly, 300 μl cell suspension and 500 μl C-peptide-containing growth medium were added to the collagen-coated insert and lower chamber, respectively. After 24 h of incubation, non-invading cells were removed using cotton swabs and processed according to the manufacturer’s protocol. The number of invading cells was quantified by measuring the optical density at 560 nm.

### Nuclear Fractionation

UnD-ESCs were grown in a 6 cm plate to 100% confluence in the growth medium, serum-starved for 48 h, and treated with C-peptide (50 nM) for 10 min. Pretreatment with Akti (1 µM) and okadaic acid (OA) (30 nM) for 1 h was performed according to the figure legends. The cells were then washed three times using cold PBS, lysed in 300 µL cytoplasmic protein extraction buffer [5 mM KCl, 5 mM HEPES, 0.05 mM ethylene glycol-bis (β-aminoethyl ether)-N,N‘,N′,N-tetraacetic acid (EGTA), 0.05 mM EDTA, and 0.075% NP-40], and incubated on ice for 30 min, with shaking every 10 min. After centrifugation at 1500 × *g* for 10 min at 4°C, the supernatant was then transferred into a new tube and stored as a cytoplasmic extract. The remaining nuclear pellet was washed three times using cytoplasmic extraction buffer, re-suspended in 100 μl nuclear protein extraction buffer (0.25 M EDTA, 0.5 M Tris-HCl pH 7.4, 2.5 M NaCl, 5% sodium deoxycholate, 5% SDS, and 5% Triton X-100), and then incubated on ice for 30 min, with shaking after every 10 min. The nuclear extracts were collected by centrifugation at 13,000 × *g* for 30 min at 4°C. Both the cytoplasmic and nuclear extracts were boiled for 5 min in 5X SDS sample buffer and subjected to western blot analysis.

### Lentivirus-Mediated Short Hairpin RNA

The shRNA clone for β-catenin (CTNNB1) was obtained from Sigma-Aldrich in the pLKO.1-puro vector (MISSION shRNA). The clone IDs were TRCN0000314991 and TRCN0000314921 for shCTNNB1-1 and shCTNNB1-2, respectively. The shRNA clones for protein phosphatase catalytic subunit a (PPP1Ca) and scramble control have been previously reported (Khaliq et al., 2020). Lentivirus packaging and testing were performed as described previously (Baek et al., 2018).

### Protein Phosphatase Assay

Protein phosphatase activity was assayed as previously described (McAvoy and Nairn, 2010; Khaliq et al., 2020). Briefly, UnD-ESCs were lysed in passive lysis buffer (Promega) and reacted with p-nitrophenylphosphate for 45 min in a colorimetric assay buffer (20 mM Tris pH 7.5, 5 mM MgCl_2_, 1 mM EGTA, 0.02% β-mercaptoethanol, and 0.1% bovine serum albumin). Absorbance was measured at 405 nm wavelength. The lysate containing the phosphatase inhibitor was used as a blank.

### Zymography

Gelatin zymography was performed as previously reported (Toth and Fridman, 2001). Briefly, UnD-ESCs were grown to 100% confluence in the growth medium, serum-starved for 48 h, and treated with 50 nM C-peptide for 24 h. The media was collected, centrifuged (400 ×*g*, 5 min at 4°C) to remove cells and debris, and concentrated to 500 μl using an Amicon^®^ Ultra-15 Centrifugal Filter Unit (UFC901024, Merck, Darmstadt, Germany). Then, 500 μl of clarified conditioned media was mixed with 30 μl of 50% slurry of gelatin–agarose beads (6025-10, Adar Biotech, Rehovot, Israel) and rotated at 4°C for 3 h. The resulting beads were washed twice using TBS-B (50 mM Tris-HCl, pH 7.5, 150 mM NaCl, 5 mM CaCl_2_, and 0.02% Brij-35) and boiled with 60 μl of 1 × SDS sample buffer for 5 min. The reaction mixture was analyzed using 10% SDS-polyacrylamide with 0.1% gelatin gel electrophoresis. The gel was incubated with renaturing solution (2.5% v/v Triton X-100) for 30 min and developing buffer (50 mM Tris-HCl, pH 7.8, 0.2 M NaCl, 5 mM CaCl_2_, and 0.02% Brij-35) for 30 min with gentle agitation and subsequently with fresh developing buffer at 37°C for 16 h. The gel was stained with staining solution (0.5% Coomassie blue R-250, 5% methanol, and 10% acetic acid in dH_2_O) for 1 h and then destained with destaining solution (10% methanol, 5% acetic acid in dH_2_O) until areas of gelatinolytic activity appeared as clear sharp bands over the blue background. The intensity of MMP2/9 was measured using ImageJ. Each MMP9 level (monomers and dimers) was calculated by normalization with MMP2 levels and then dividing by the total MMP9 level.

### Chromosome Immunoprecipitation-PCR Assay

ChIP was performed according to the manufacturer’s protocol (Abcam, Cambridge, United Kingdom). Briefly, cells were incubated with 0.75% formaldehyde for 30 min and disrupted by sonication at 20 Hz in ChIP lysis buffer [50 mM Tris-HCl, pH 7.4, 1% Igepal CA-630 (I3021, Sigma)], 0.25% sodium deoxycholate, 150 mM NaCl, 1 mM EDTA, 0.1% SDS, 0.5 mM dithiothreitol (DTT), 5 mM sodium butyrate, protease inhibitor cocktail (P8340, Sigma), phosphatase inhibitor cocktail I (P2850, Sigma), and phosphatase inhibitor cocktail II (P5726, Sigma). Nuclei were harvested and disrupted by sonication at 20 Hz. After centrifugation at 14,000 × *g* for 10 min, the supernatant was incubated overnight with anti-β-catenin (8480S, Cell Signaling Technology) and precipitated using protein A/G-PLUS-agarose beads (SC-2003, Santa Cruz Biotechnology, Dallas, TX, United States). The resulting beads were washed twice with ChIP wash buffer (I-IV; Supplementary Table S2) 2 times with each buffer, and the bound DNA was eluted in elution buffer (1% SDS and 100 mM NaHCO_3_). Finally, RNA and proteins in the eluted solutions were removed using RNase and proteinase K, respectively, and the DNA was further purified using phenol-chloroform extraction. The resulting DNA was used for PCR analysis using specific primers for the putative binding site of β-catenin/TCF1 in MMP9, TIMP1, and TIMP3 (Supplementary Table S3).

### Statistical Analysis

Data are presented as the mean ± standard deviation (SD) of at least three independent experiments (three to six independent experiments for all figures). All individual data points were represented using dots in all the quantified graphs. Where necessary, the statistical significance of the data was determined using a two-tailed paired Student’s *t*-test in Excel or GraphPad Prism version 9.0 for Microsoft Windows (GraphPad Software, La Jolla, CA, United States). Statistical significance was set at *p* < 0.05.

## Results

### C-Peptide Promotes Cell Migration in Human UnD-ESCs and Jeg3 Cells

**FIGURE 1:**
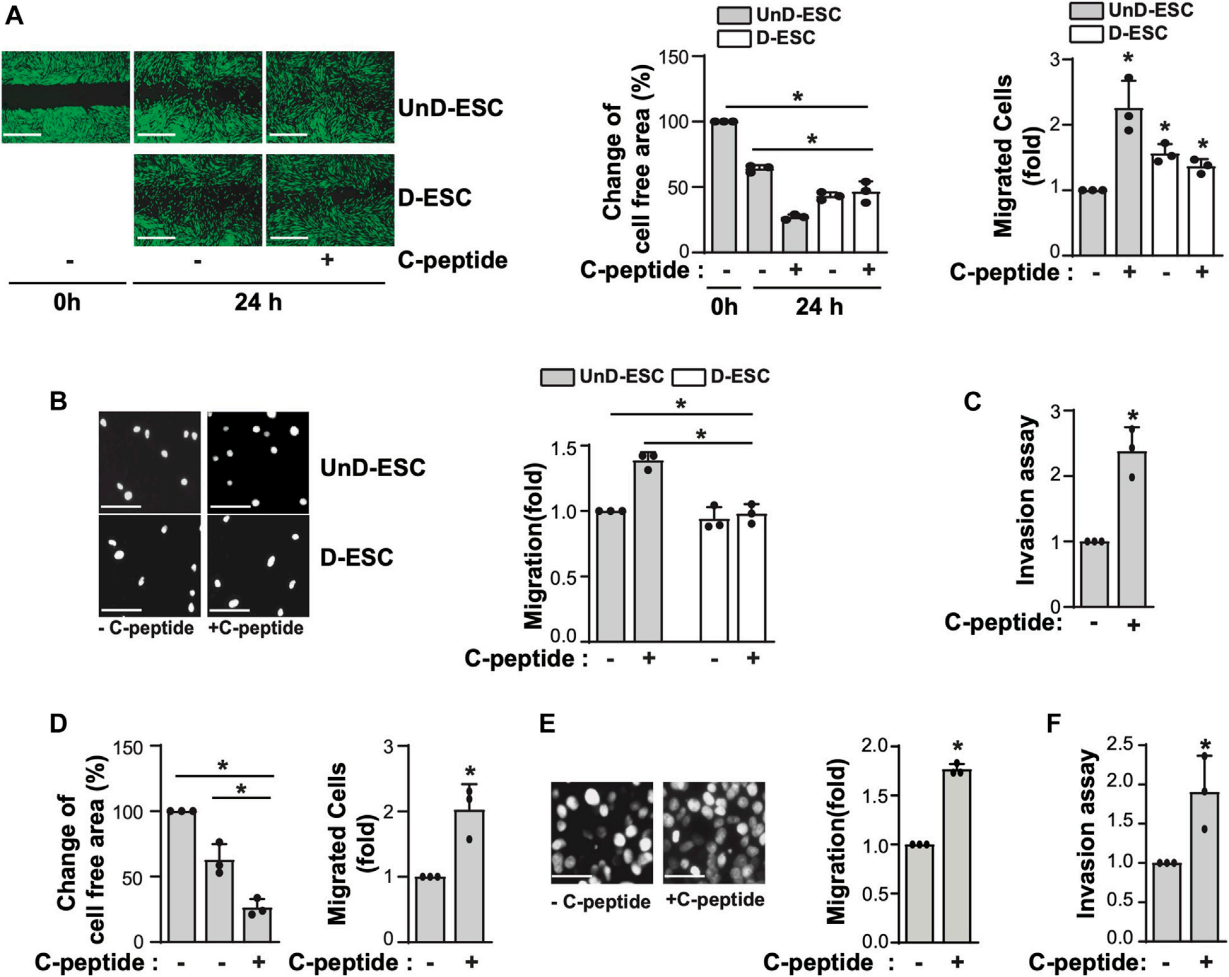
C-peptide enhances cell migration and invasion in undecidualized human endometrial stromal cells (UnD-ESCs) and Jeg3 cells. **(A–D)** The isolated HESCs were used as the undecidualized HESCs (UnD-ESCs) and decidualized HESCs (D-ESCs) on day 2 after the induction of decidualization in the presence of 8-Br-cAMP. **(A)** UnD-ESCs or D-ESCs were serum-starved for 18 h, wounded using a T200 tip, and incubated with or without 50 nM C-peptide for 24 h. Then, HESCs were stained using the CytoPainter Cell Tracking Staining Kit, and visualized using an Olympus CKX53 microscope (X4). **(B)** UnD-ESCs or D-ESCs were plated on trans-well filters and 50 nM C-peptide was added to the outer chamber of each filter. The cells were incubated for 24 h and stained using DAPI and visualized using an Olympus CKX53 microscope (X4). **(C)** UnD-ESCs were split on collagen-coated trans-wells and treated as described in **(B)**, following the manufacturer’s instruction. **(D)** HESCs from three participants (n = 3) were subjected to wound-healing scratch assays for 24 h, with or without 50 nM C-peptide. **(E)** JEG-3 cells were treated and analyzed as described in **(B)**. **(F)** JEG-3 cells were treated and processed as described in **(C)**. Asterisks (*) indicate significant differences (*p* < 0.05) when compared to C-peptide untreated control or C-peptide treated UnD-ESCs control for 24 h. Scale bar = 100 μm.

We first examined the effect of C-peptide on cell migration in both UnD-ESCs and D-ESCs. D-ESCs were used after UnD-ESCs were incubated with 8-Br-cAMP for 2 days to induce decidualization. When both cells were subjected to a wound-healing scratch assay in the presence or absence of C-peptide, the D-ESCs migrated faster than the UnD-ESCs (Figure 1A), consistent with a previous report (Gellersen et al., 2010). Notably, C-peptide treatment enhanced the motility of UnD-ESCs, as indicated by the lower cell-free area and a greater number of migrated cells but did not change the motility of D-ESCs (Figure 1A). Both two different concentrations of C-peptide, 1 nM as well as 50 nM, increased cell motility in UnD-ESCs (Supplementary Figure S1 and Figure 1A). Transwell and invasion assays were used to further examine the differential migration of UnD-ESCs and D-ESCs. Indeed, C-peptide treatment significantly increased the motility of UnD-ESCs but not that of D-ESCs in the Transwell assay (Figure 1B) and enhanced the invasion of UnD-ESCs in collagen-coated transwell filters (Figure 1C), in agreement with the results of the *in vitro* wound-healing scratch assay. Furthermore, the effect of C-peptide on cellular motility was confirmed using primary human ESCs from three individuals, implying that faster cell migration by C-peptide treatment is universal in human ESCs (Figure 1D). To further investigate whether C-peptide regulates trophoblast migration and invasion, the migration of trophoblast-derived JEG-3 cells was assessed using a Transwell assay and an invasion assay. C-peptide increased the rate of JEG-3 cell migration in the Transwell (Figure 1E) and the number of invaded JEG-3 cells in the collagen-coated transwell filters (Figure 1F). These results suggest that C-peptide has a profound positive effect on the migration of UnD-ESCs and JEG-3 cells.

### C-Peptide Controls β-Catenin Phosphorylation and Nuclear Localization in an Akt and Protein Phosphatase-Dependent Manner

**FIGURE 2:**
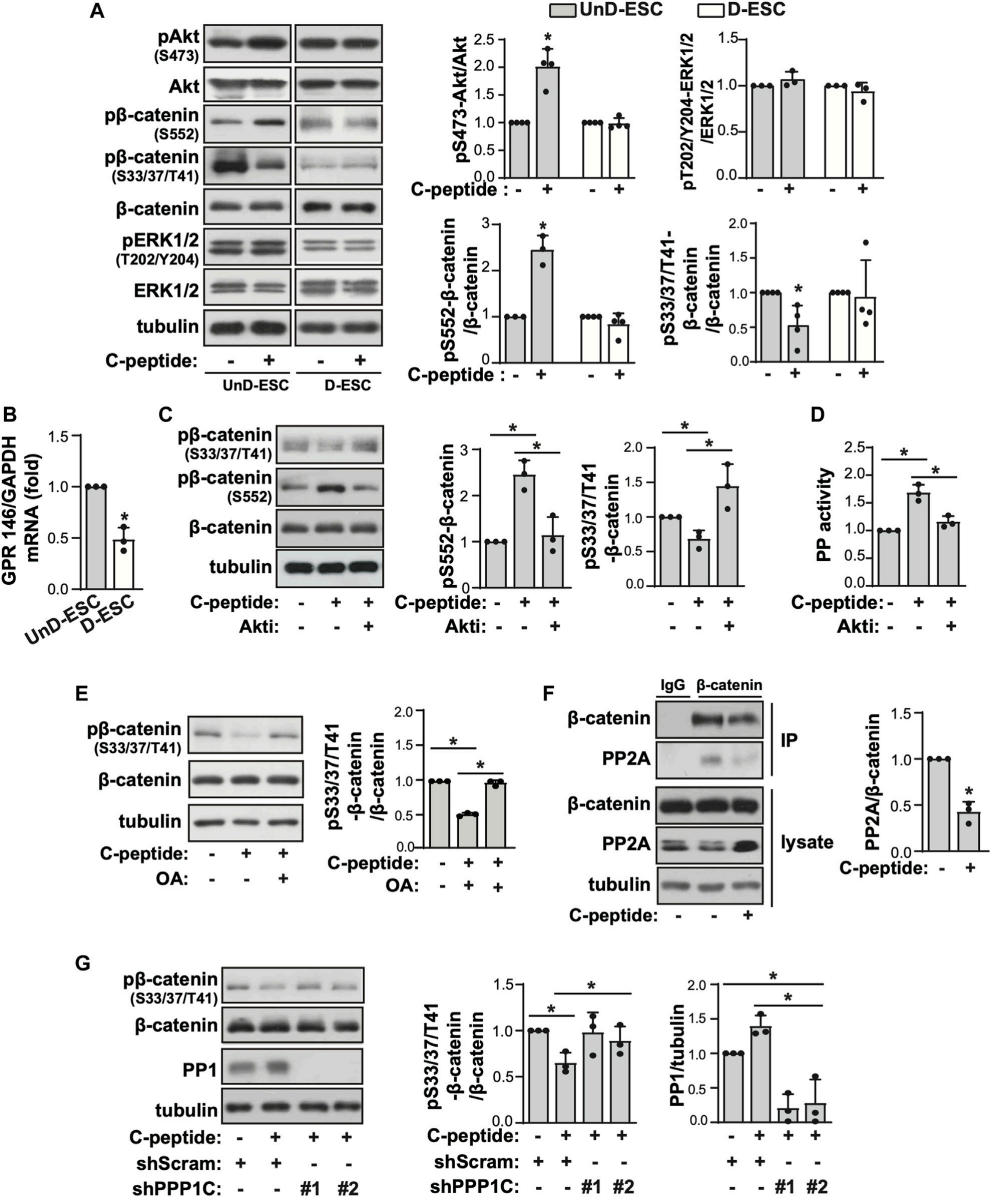
Akt and PP control C-peptide-induced β-catenin phosphorylation. **(A)** The isolated human endometrial stromal cells (HESCs) were used as the undecidualized HESCs (UnD-ESCs) and decidualized HESCs (D-ESCs) on day 2 after the induction of decidualization in the presence of 8-Br-cAMP. UnD-ESCs and D-ESCs were serum-starved for 48 h and then incubated with 50 nM C-peptide for 10 min. The cells were lysed and subjected to western blot analysis. **(B)** UnD-ESCs and D-ESCs were lysed and GPR146 mRNA level was analyzed using qRT-PCR. **(C)** UnD-ESCs were pretreated with 1 μM Akti for 1 h and treated as described in **(A)**. **(D)** UnD-ESCs were treated as described in **(C)** and protein phosphatase (PP) activity was measured. **(E)** UnD-ESCs were treated as described in **(A)** in pretreatment with 10 μM okadaic acid for 1 h. **(F)** After treatment with C-peptide for 10 min, UnD-ESCs were lysed and then immunoprecipitated with IgG or anti-β-catenin. The PP2A levels of the immunoprecipitates were normalized using the β-catenin level. **(G)** UnD-ESCs were transduced using short hairpin RNAs (shRNAs), selected for 5 days with 1.5 μM puromycin and treated as **(A)**. **(A,C,E,G)** The intensities of western blots were quantified using ImageJ software. The relative phosphorylation level or protein expression level were calculated by comparing them to total protein or tubulin level, respectively. Asterisks (*) indicate significant differences (*p* < 0.05) when compared to C-peptide untreated control or C-peptide treated UnD-ESCs control. Akti, Akt inhibitor; OA, Okadaic acid; shScram, shRNA for Scramble; shPPP1C, shRNA for PPP1C.

**FIGURE 3:**
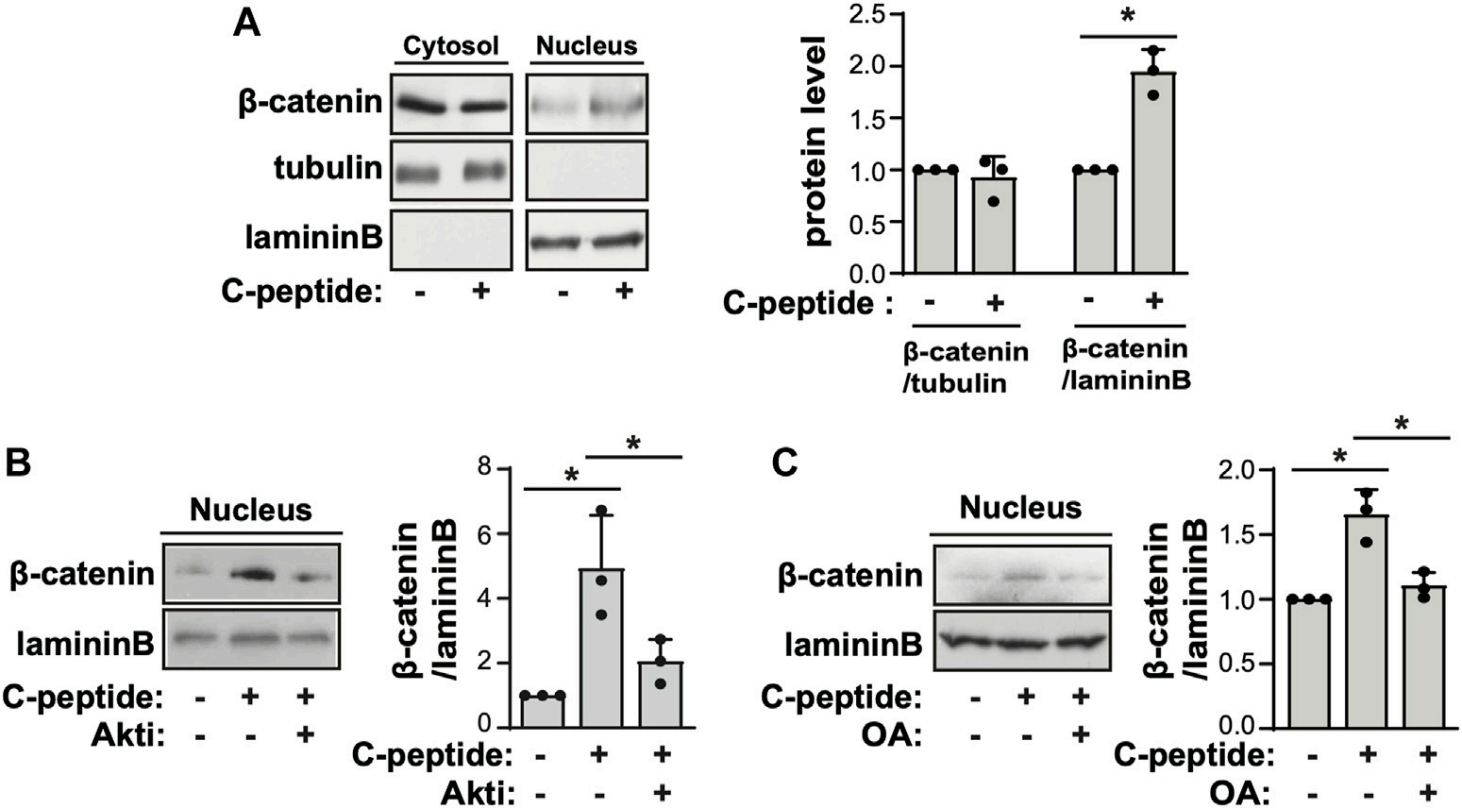
C-peptide induces β-catenin nuclear translocation in an Akt and protein phosphatase-dependent manner. **(A)** The undecidualized human endometrial stromal cells (UnD-ESCs) were serum-starved for 48 h, incubated with 50 nM C-peptide for 10 min, and later lysed using cytoplasmic protein extraction buffer. The cytoplasmic and nuclear extracts were fractionated using the cytoplasmic and nuclear protein extraction buffers, respectively. Both cell lysates were subjected to western blot analysis. Tubulin and laminin B were used as cytoplasmic and nuclear markers, respectively. **(B,C)** The cells were pretreated with 1 μΜ Akti **(B)** or 10 nM OA **(C)** and treated as described in **(A)**. Nuclear cell lysate was analyzed by western blot. The intensities of western blots were quantified using ImageJ software. The relative β-catenin level was calculated by comparing tubulin level for the cytoplasmic fraction and laminin B for the nuclear fraction, respectively. Asterisks (*) indicate significant differences (*p* < 0.05) when compared to C-peptide untreated UnD-ESCs control or C-peptide treated UnD-ESCs control. Akti, Akt inhibitor; OA, Okadaic acid.

To investigate the underlying mechanism of the differential response to C-peptide in the migration of UnD-ESCs and D-ESCs, the effects of C-peptide on the activities of ERK1/2 and Akt were examined in both UnD-ESCs and D-ESCs. C-peptide treatment significantly enhanced the phosphorylation of Akt at Ser 473, but not that of ERK1/2, in UnD-ESCs (Figure 2A). At the same time, the phosphorylation of β-catenin was increased at Ser 552 and decreased at Ser33/37/Thr41 (Figure 2A). However, the phosphorylation of Akt and β-catenin remained unchanged in D-ESCs (Figure 2A). The mRNA level of G-protein coupled receptor 146 (GPR146), a putative C-peptide receptor, was higher in UnD-ESCs than in D-ESCs (Figure 2B), indicating a stronger response to C-peptide in UnD-ESCs, although C-peptide decreased GPR146 expression slightly, but not significantly (Supplementary Figure S2). Meanwhile, pretreatment with an Akt inhibitor (Akti) reduced β-catenin phosphorylation at Ser 552, but restored phosphorylation at Ser33/37/Thr41 (Figure 2C). Notably, pretreatment with Akti dampened C-peptide induced protein phosphatase (PP) activation, whereas C-peptide augmented PP activity, as we previously reported (Figure 2D). OA, a PP inhibitor, restored β-catenin phosphorylation at Ser33/37/Thr41 (Figure 2E). These results suggest that Akt is involved in β-catenin phosphorylation at Ser 552 directly and at Ser33/37/Thr41 by regulating PP activity. To identify which PP is responsible for the dephosphorylation of β-catenin, we next determined whether C-peptide altered the interaction between β-catenin and PP2A. PR55α, a regulatory subunit of PP2A, specifically binds to β-catenin and modulates PP2A-mediated β-catenin dephosphorylation (Zhang et al., 2009; Maheshwari et al., 2017). C-peptide treatment dissociated PP2A from β-catenin (Figure 2F), suggesting that PP2A might not be responsible for β-catenin dephosphorylation at Ser33/37/Thr41. However, treatment with C-peptide did not alter β-catenin phosphorylation at Ser33/37/Thr41 in UnD-ESC-depleted catalytic subunit alpha isozyme of protein phosphatase 1 (PPP1Ca) by transduction with shRNA, whereas it reduced β-catenin phosphorylation in scrambled shRNA-transduced cells (Figure 2G). In line with previous findings that the phosphorylation of β-catenin at Ser 552 and dephosphorylation of β-catenin at Ser33/37/Thr41 stimulates the nuclear translocation and transcriptional activity of β-catenin (Fang et al., 2007; Jacobs et al., 2012), C-peptide increased nuclear β-catenin levels without a significant decrease in cytoplasmic β-catenin levels (Figure 3A). However, pretreatment with Akti and OA reduced the nuclear translocation of β-catenin (Figure 3B, Supplementary Figure S3, and Figure 3C). These results indicate that C-peptide regulates β-catenin phosphorylation and promotes the nuclear translocation of β-catenin in an Akt-or PP-dependent manner.

### C-Peptide Modulates the Expression of MMP9 and TIMP1/3 Through β-Catenin in UnD-ESCs

**FIGURE 4:**
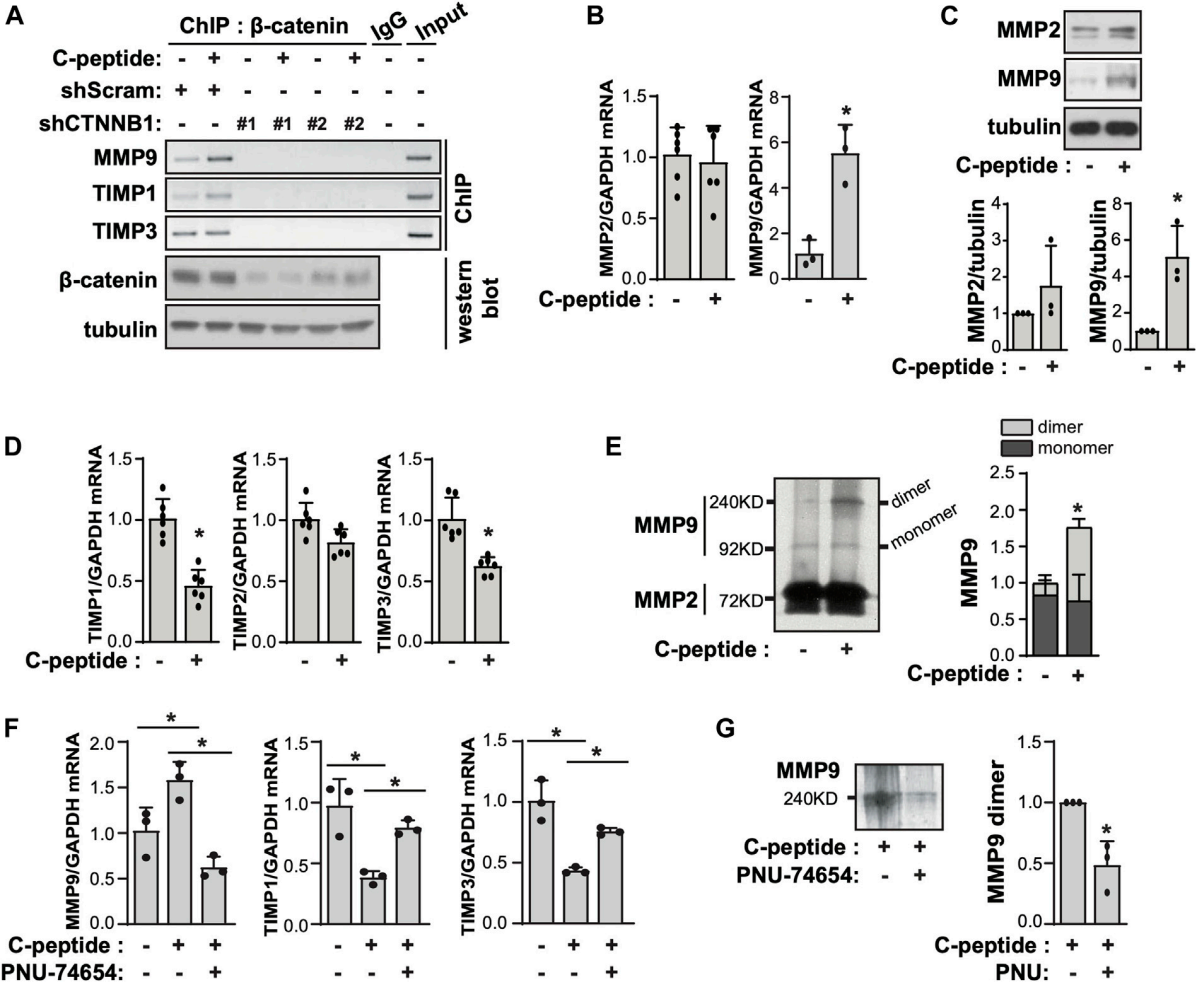
C-peptide increases the expression of MMP9/TIMP1/TIMP3 and the activity of MMP9 in a β-catenin-dependent manner in undecidualized human endometrial stromal cells. **(A)** The undecidualized human endometrial stromal cells (UnD-ESCs) were transduced using short hairpin RNAs (shRNAs) for scramble and β-catenin and selected for 5 days with 1.5 μM puromycin. Then, cells were serum-starved for 48 h and treated with 50 nM C-peptide for 24 h. UnD-ESCs were lysed and subjected to chromatin immunoprecipitation. The knockdown of β-catenin was confirmed using western blot. **(B)** UnD-ESCs were serum-starved for 48 h and then treated with 50 nM C-peptide for 24 h. The cell lysates were analyzed using qRT-PCR for MMP2, MMP9, and GAPDH. **(C)** UnD-ESCs were treated as described in **(B)**, lysed, and then subjected to western blot analysis. **(D)** UnD-ESCs were treated as described in **(B)**, and subjected to qRT-PCR for TIMP1, TIMP2, TIMP3, and GAPDH **(E)** UnD-ESCs were serum-starved for 48 h and then incubated with or without 50 nM C-peptide for 24 h. The cell medium was collected and analyzed using gelatin zymography. The relative MMP9 activity was calculated by measuring the intensities of MMPs band using ImageJ software. **(F)** UnD-ESCs were serum-starved for 48 h, pretreated with 10 μM PNU-74654 for 1 h, and then incubated with or without 50 nM C-peptide for 24 h. The cells were lysed and subjected to qRT-PCR analysis for MMP9, TIMP1, TIMP3, and GAPDH. **(G)** UnD-ESCs were treated as **(F)** and the cell medium was collected and subjected to gelatin zymography. Asterisks (*) indicate significant differences (*p* < 0.05) when compared to C-peptide untreated UnD-ESCs control or C-peptide treated UnD-ESCs control. shScram, shRNA for Scramble; shCTNNB1, shRNA for β-catenin.

When β-catenin is translocated into the nucleus, it directly interacts with TCF/LEF to regulate the expression of target genes (Zhang and Yan, 2016). We found that the putative binding site of TCF-1 was present in the proximal promoter regions of MMP9, TIMP1, and TIMP3, which are important for cellular movement through the extracellular matrix (ECM) (Supplementary Figure S4). To probe the potential role of β-catenin in transcriptional regulation of the expression of MMP9, TIMP1, and TIMP3 in UnD-ESCs, we performed a ChIP-PCR assay with anti-β-catenin. As shown in Figure 4A, C-peptide treatment significantly increased the binding of β-catenin to the putative binding regions of MMP9, TIMP1, and TIMP3. Meanwhile, β-catenin knockdown by lentiviral shRNA targeting β-catenin almost completely blocked these bindings, suggesting that β-catenin functions as a transcriptional regulator of MMP9, TIMP1, and TIMP3 (Figure 4A). Consistently, C-peptide treatment significantly enhanced the mRNA and protein levels of MMP9, but not that of MMP2 (Figures 4B,C), whereas it significantly reduced the mRNA expression of TIMP1 and TIMP3, but not that of TIMP2 (Figure 4D). MMP-9 is secreted in a latent form, pro-MMP-9, which is glycosylated and contains an additional proline-rich insertion (54 amino acids) between the catalytic and hemopexin-like domains (Collier et al., 1988). The glycosylated mature pro-MMP-9 monomer was found to be ∼92 kDa in zymography gel, whereas the pro-MMP-9 dimer was ∼240 KD (Sclafani and Wendell, 2001; Pino et al., 2009). Notably, the activity of pro-MMP9 dimers was increased in conditioned media of C-peptide-treated cells, as demonstrated by gelatin zymography analysis (Figure 4E), implying that the changes in the expression of MMP9, TIMP1, and TIMP3 could increase the level of pro-MMP-9 dimers and pro-MMP9 activity.

To examine the involvement of β-catenin in MMP9 expression and activity in UnD-ESCs, we pretreated UnD-ESCs with PNU-74654, a β-catenin inhibitor, in C-peptide-treated UnD-ESCs. PNU-74654 significantly reduced MMP9 expression and restored TIMP1 and TIMP3 expression in C-peptide-treated UnD-ESCs (Figure 4F), subsequently resulting in decreased MMP 9 dimers activity as demonstrated by gelatin zymography analysis (Figure 4G).

### β-Catenin is Indispensable for C-Peptide-Induced Migration of UnD-ESCs by Regulating MMP9 Activity

**FIGURE 5:**
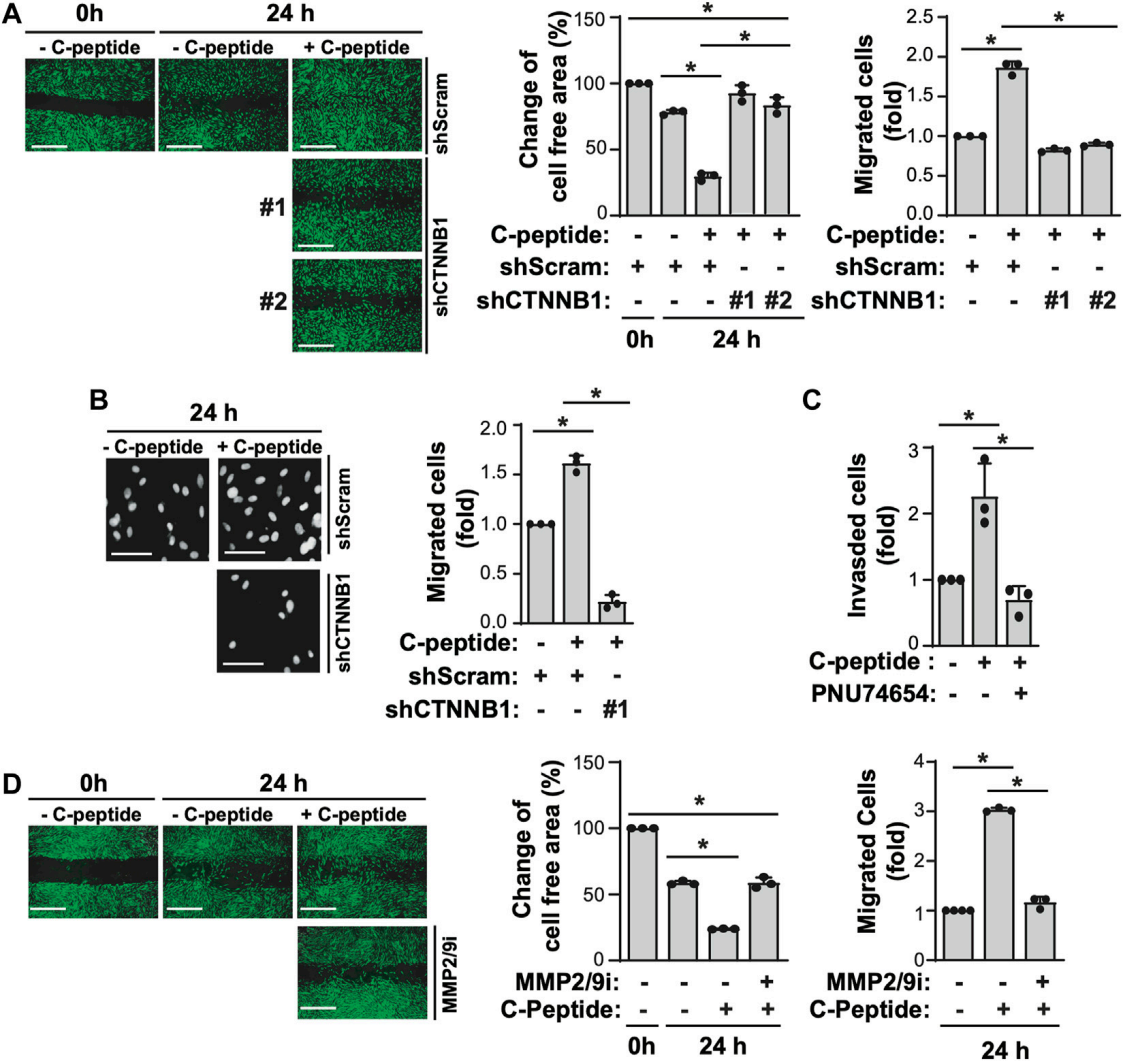
β-catenin and MMP9 are critical for C-peptide-induced migration of undecidualized human endometrial stromal cells. **(A)** Undecidualized human endometrial stromal cells (UnD-ESCs) were transduced using shRNAs for scramble and β-catenin and selected for 5 days with 1.5 μM puromycin. The cells were serum-starved for 18 h, wounded using a T200 tip, incubated for 24 h with or without 50 nM C-peptide, visualized using the CytoPainter Cell Tracking Staining Kit and an Olympus CKX53 microscope (X4), and finally analyzed using ImageJ software. **(B)** UnD-ESCs were introduced with shRNAs and selected as described in **(A)**, and then split on transwell filters. C-peptide (50 nM) was added to the outer chamber of each filter. The cells were incubated for 24 h, stained with DAPI, and visualized using an Olympus CKX53 microscope (X4). **(C)** UnD-ESCs were split on collagen-coated trans-wells, pretreated with 10 μM PNU-74654 for 1 h, and then incubated for 24 h with or without 50 nM C-peptide, following the manufacturer’s instructions. **(D)** UnD-ESCs were serum-starved for 18 h, wounded using a T200 tip, pretreated with 10 μM MMP2/9 inhibitor for 1 h, and then incubated with or without 50 nM C-peptide for 24 h. The cells were visualized using the CytoPainter Cell Tracking Staining Kit and an Olympus CKX53 microscope (X4), and then analyzed using ImageJ. Asterisks (*) indicate significant differences (*p* < 0.05) when compared to the C-peptide-untreated UnD-ESCs control or C-peptide-treated UnD-ESCs control. Scale bar = 100 μm shScram, shRNA for scramble; shCTNNB1, shRNA for β-catenin; MMP2/9i, MMP2/MMP9 inhibitor.

**FIGURE 6:**
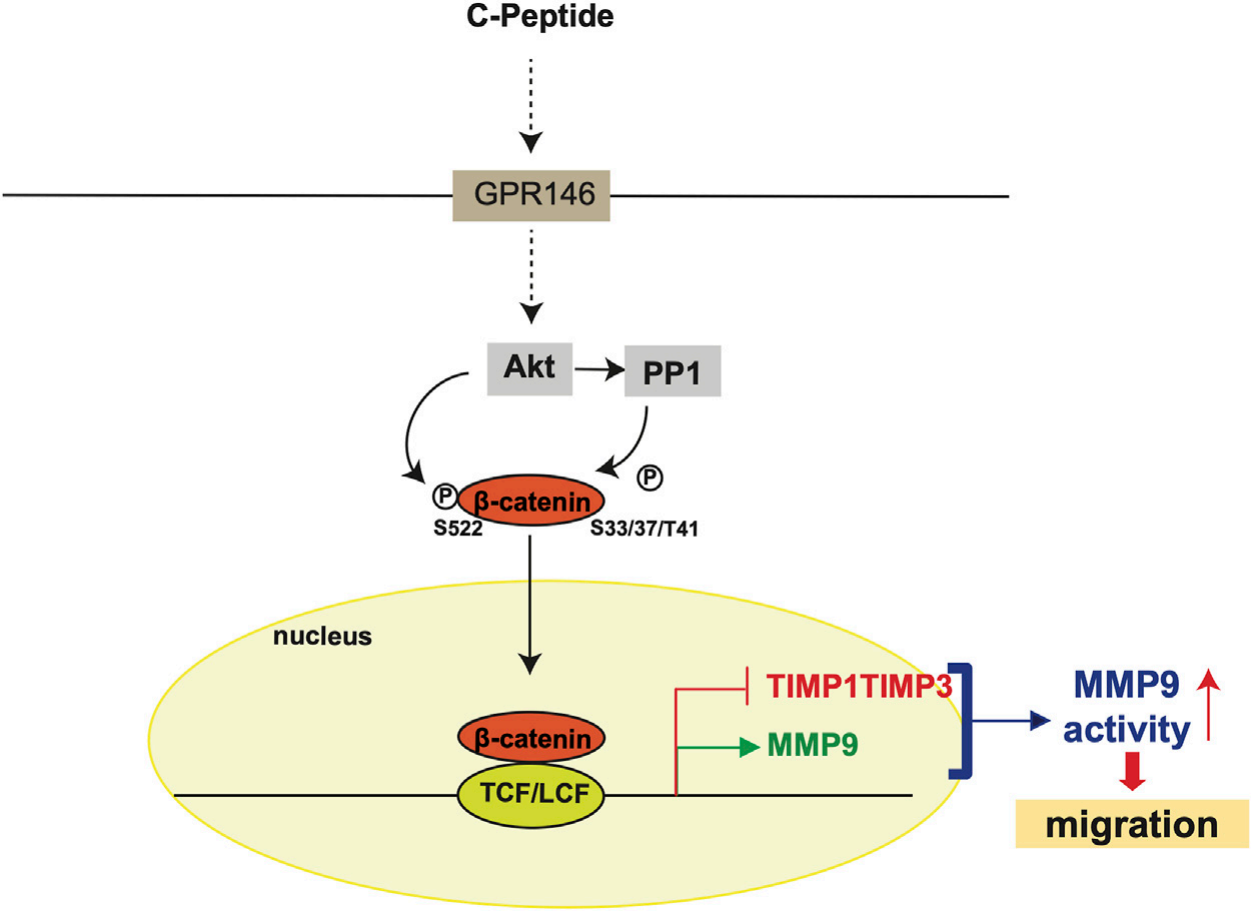
Hypothetical model of C-peptide-enhanced migration in undecidualized human endometrial stromal cells. C-peptide activates Akt, which may occur through GPR146, in undecidualized human endometrial stromal cells (UnD-ESCs). Activated Akt phosphorylates β-catenin at Ser522 and dephosphorylates β-catenin at Ser33/37/Thr41 by activating PP1. β-catenin is then translocated to the nucleus where β-catenin/TCF-1/LEF binds to the proximal promoter regions of MMP9, TIMP1, and TIMP3. The upregulation of MMP9 expression and downregulation of TIMP1 and TIMP3 expression enhanced MMP9 activity, resulting in enhanced cell migration in UnD-ESCs.

The observation that β-catenin plays a critical role in MMP9 activity by modulating the expression of MMP9/TIMP1/TIMP3 led us to test the effect of β-catenin on C-peptide-induced migration in UnD-ESCs**.** β-catenin knockdown by delivering lentivirus shRNA for β-catenin significantly reduced UnD-ESC migration, as demonstrated by wound healing scratch assay and Transwell assay (Figures 5A,B, and Supplementary Figure S3A). Consistent with this, β-catenin inhibition by PNU-74654 pretreatment also blocked C-peptide-stimulated cell motility of UnD-ESCs in the wound healing scratch (Supplementary Figure S3B) and Transwell assay (Supplementary Figure S3C). In addition, invasion of the collagen-coated trans-well filters was inhibited in UnD-ESCs pretreated with PNU-74654 (Figure 5C). In line with the essential role of β-catenin in UnD-ESC migration, the inhibition of upstream regulators of β-catenin by pretreatment with Akti or OA as well as PPP1Ca knockdown also decreased C-peptide-induced UnD-ESC migration, as shown by the wound healing scratch (Supplementary Figures S4A,B) and Transwell assay (Supplementary Figure S4C). In addition, pretreatment with the MMP9 inhibitor completely inhibited C-peptide-enhanced migration in UnD-ESCs by a wound healing scratch assay (Figure 5D), suggesting that MMP9 is critical for C-peptide-induced migration in UnD-ESCs. Taken together, these results suggest that C-peptide augments the activity and the expression of MMP9 and decreases the expression of TIMP1 and TIMP3 through the direct regulation of β-catenin, thereby controlling cell migration in UnD-ESCs (Figure 6).

## Discussion

The migration and invasion of ESCs are required for crosstalk between the decidua and trophoblasts at the fetal-maternal interface (Gellersen et al., 2010). D-ESCs respond to local stimuli from trophoblasts and move to trophoblasts, and vice versa. The migration of D-ESCs is faster than that of UnD-ESCs, facilitating decidua-trophoblast communication (Gellersen et al., 2010). Thus, the disturbance of cell mobility rate between D-ESCs and UnD-ESCs might be detrimental to the success of implantation by blocking the interaction between trophoblasts and D-ESCs. Here, we demonstrate that C-peptide preferentially enhances cell migration and invasion of UnD-ESCs by regulating MMP9 activity through β-catenin (Figure 6); C-peptide in UnD-ESCs induces nuclear translocation of β-catenin through regulation by Akt and PP1, and β-catenin in the nucleus directly regulates the transcription of MMP9/TIMP1/TIMP3. An increase in MMP9 expression and a decrease in the expression of TIMP1 and TIMP3 augments MMP9 activity, resulting in faster migration of UnD-ESCs. Our results suggest that C-peptide levels are critical for successful fetal-maternal interfaces.

β-catenin is a downstream effector of canonical Wingless and Int-1 (WNT) signaling, which regulates cell adhesion junctions and target gene expression (Patterson et al., 2017). The function of β-catenin in endometrial processes, mainly decidualization and implantation, has been suggested in previous studies (Mohamed et al., 2004; Deutscher and Hung-Chang Yao, 2007; Zhang and Yan, 2016). Although β-catenin does not significantly affect blastocyst formation (Mohamed et al., 2004), uterine β-catenin is required for successful implantation (Zhang and Yan, 2016) and uterine differentiation (Deutscher and Hung-Chang Yao, 2007). Deficiency of β-catenin in mice showed further sub-fertility through improper STAT signaling in stromal and epithelial cells during embryo implantation and decidualization (Patterson et al., 2017). The interaction between EHD1 and WNT4 blocks WNT4/β-catenin signaling, thereby impairing decidualization and causing recurrent implantation failure (Zhou et al., 2019). Recently, the abnormal activation of β-catenin in the uterus has been reported to activate TGF-β signaling and thus cause adenomyosis through epithelial–mesenchymal transition (Yoo et al., 2020). Consistent with the increased invasiveness of aberrant β-catenin-activated cells, the present study demonstrates that abnormal β-catenin activation enhances cell migration and invasion through direct transcriptional regulation of MMP9 and TIMP1/3 expression, following an increase in MMP9 activity in the presence of C-peptide. Therefore, β-catenin activation in UnD-ESCs could be tightly repressed under normal levels of C-peptide, thereby maintaining the levels of MMP9 and TIMP1/3 expression and low cellular motility.

MMP9 has been reported as a major regulator of cellular migration and invasion that functions through the degradation of the ECM (Fluhr et al., 2008; Liu et al., 2013; Liu et al., 2019). The involvement of β-catenin in the C-peptide-enhanced MMP9/TIMP1 ratio and migration is consistent with the fact that β-catenin directly regulates the MMP9 promoter during endometriosis, which shows high potential for migration, and β-catenin and MMP9 increase in the impaired decidualization of isolated human ESCs from patients with endometriosis (Zhang et al., 2016). Pro-MMP-9 monomer forms pro-MMP9 homodimers by covalently tethering the hemopexin domain of MMP-9 *via* a disulfide bond (Olson et al., 2000). This is a unique feature of MMP-9, where pro-MMP9 dimerization is required for MMP-9-increased cell migration (Dufour et al., 2010). The activity of MMP-9 is modulated by interactions with TIMP1; TIMP1 forms a non-covalent complex with MMP9 *via* the C-terminal PEX domain of MMP9, whereas the N-terminus of TIMP1 is open to inhibit active MMPs (Gellersen et al., 2010). TIMP1 inhibits MMP-9 dimerization by blocking the PEX domain of MMP-9, resulting in decreased cell migration (Dufour et al., 2010). Hence, the ratio of MMP9/TIMP1 and the level of secreted pro-MMP-9 are determined by the invasiveness of cells or tissues. This finding is supported by a positive correlation between the ratio of MMP9/TIMP1 in the endometrial tissue of patients with endometriosis and the ectopic development of the disease and an increase in pro-MMP-9 dimer levels in endometriosis patients (Collette et al., 2006; Pino et al., 2009). In the current study, an increase in pro-MMP-9 dimer levels is a critical cue to drive cell migration in UnD-ESCs. In addition, TIMP3 appears to function mostly as a metalloproteinase inhibitor to regulate the extracellular matrix (Murphy, 2011). Although C-peptide decreased TIMP3 expression in a β-catenin-dependent manner (Figure 4), it did not substantially enhance the interaction of β-catenin with the transcriptional regulatory domain of TIMP3, suggesting that C-peptide might indirectly regulate TIMP3 expression in a β-catenin-dependent manner. In addition to changes in the interaction of β-catenin with the transcriptional regulatory domain of target genes, posttranslational modifications of β-catenin or TCF-1 are considered important determinants of target gene transcriptional repression. Trimethylation of β-catenin at Lys49 increases the repression of genes related to embryonic stem cell pluripotency (Hoffmeyer et al., 2017). The epigenetic modification of TCF-1 represses interleukin (IL)-17 during T cell development (Ma et al., 2011). However, TCF-1 drives the modulation of the chromatin state, promoting the sequential suppression of MAF and RORγt, which subsequently controls CD8^+^ T cell fate decisions in double-positive thymocytes (Mielke et al., 2019). Therefore, it is possible that C-peptide induces epigenetic modifications of either β-catenin or TCF-1 to repress TIMP3; hence, further investigation is warranted.

The level of C-peptide in patients with PCOS is 0.77–1.69 nM under fasting conditions (O'Meara et al., 1993; de Medeiros et al., 2018). In healthy controls, the level is 0.3–0.6 nM under fasting conditions and 1–3 nM under postprandial conditions (Leighton et al., 2017). The C-peptide level is higher in diabetes patients (3.33 ng/ml; 0.874 nM) than in normal subjects (1.79 ng/ml; 0.592 nM) (Donatelli et al., 1991). In the present study, we tested the effect of 1 and 50 nM C-peptide on cell migration (Supplementary Figure S1 and Figure 1A). A C-peptide concentration of 1 nM is representative of the physiological concentration of C-peptide in PCOS or diabetes patients. Both concentrations were sufficiently effective in enhancing cell migration. However, considering the short lifespan of C-peptide (half-life of 20–30 min), we utilized 50 nM C-peptide for most experiments to maintain a functional level of C-peptide for 24 h (Jones and Hattersley, 2013). In addition, we previously found that a high C-peptide concentration is required to maintain an adequate level of C-peptide for more than 24 h (Khaliq et al., 2020). Similarly, insulin was used at concentrations of 5–500 nM in experiments with human ESCs (Ujvari et al., 2017; Ujvari et al., 2018), even though its physiological concentration is 0.1 nM (Conover et al., 1994).

Insulin and C-peptide are produced in equimolar amounts by pancreatic β-cells, and insulin might be present along with C-peptide in the endometrium. Insulin enhances migration in other cells *via* signaling pathways different from those involved in the action of C-peptide; it increases motility in PCa cells, a type of human prostate cancer cell, through regulation of PI3K-MAPK (Sarkar et al., 2019) and in the oncogenic KRAS^G12D^ variant of immortalized human pancreatic duct-derived cells through MMP2 gelatinolytic activity (Wang et al., 2019). In addition, we observed that the cellular signaling induced by C-peptide is different from that induced by insulin in the endometrium (Khaliq et al., 2020). Hence, further investigation is required to assess the effect of insulin on migration in UnD-ESCs, although insulin has been shown to not affect migration in D-ESCs (Hirschberg et al., 2021).

In the present study, GPR146 mRNA levels were correlated with the degree of the response to C-peptide (Figure 2), implying that GPR146 might function as a C-peptide receptor to activate cell migration in UnD-ESCs. However, a recent study suggested the presence of an alternative C-peptide receptor instead of GPR146; GPR146 has not been confirmed as a C-peptide receptor when GPR146 was analyzed using dynamic mass distribution, an arrestin binding assay, and cellular binding and internalization under a fluorescent microscope (Lindfors et al., 2020). Meanwhile, C-peptide regulates the function of downstream effectors *via* direct binding, and binds to the breast cancer type 2 susceptibility protein (BRAC2) in mammalian zygotes in response to fertilization stimulus, and causes changes in the subcellular localization of BRAC2 to the nucleus, inducing aberrant meiotic resumption and perturbation of the DNA repair system (Martin et al., 2019). C-peptide might also directly bind to and activate the downstream effector proteins Akt or PP1. Hence, further investigation is warranted to identify the G protein-coupled receptors in UnD-ESCs and to test the possibility of C-peptide direct binding to the downstream regulators in C-peptide-induced migration.

In the present study, we found that Akt regulates β-catenin phosphorylation in two different ways: by adding phosphorylation at Ser 552 directly, and by inducing dephosphorylation at Ser33/37/Thr41 by activating PP1. Phosphorylation of β-catenin at Ser 552 or dephosphorylation of β-catenin at Ser33/37/Thr41 has been reported to stimulate its nuclear translocation and transcriptional activity (Fang et al., 2007; Jacobs et al., 2012), implying the involvement of changes in β-catenin phosphorylation in β-catenin nuclear localization. Since β-catenin phosphorylation at Ser33/37/Thr41 by GSK3β leads to its exposure to E3 ligases (Ikeda et al., 1998), C-peptide might enhance β-catenin stability by decreasing phosphorylation at Ser33/37/Thr41. In addition, β-catenin phosphorylation at Ser675 by PKA and at Tyr654 by receptor tyrosine kinase helps maintain its stability by preventing ubiquitination and reducing its interaction with E-cadherin, respectively (Hino et al., 2005; van Veelen et al., 2011). Hence, the impact of β-catenin phosphorylation, at locations other than Ser 552 and Ser33/37/Thr41, on its stability or involvement in migration merits additional exploration. Notably, we found that Akt enhanced C-peptide-induced PP activity. Akt phosphorylates PPP1R3G, which is a regulatory subunit of PP1 in insulin stimulation (Li et al., 2019). This leads to an enhancement in the binding of phosphorylated PPP1R3G for phosphorylated glycogen synthase to promote dephosphorylation of phosphorylated glycogen synthase. Whether C-peptide activated Akt increases PPP1R3G phosphorylation, followed by an increase in the binding of PPP1R3G to β-catenin remains to be investigated. In addition, we previously found that C-peptide activates PP1 without affecting Akt activity, resulting in decreased inhibitory phosphorylation of glycogen synthase kinase (GSK)3 during decidualization (Khaliq et al., 2020). Hence, we believe that PP1 is differently regulated in UnD-ESCs and D-ESCs (modulated by Akt in UnD-ESCs and by GSK-3β in D-ESCs).

Taken together, we demonstrated that C-peptide enhanced the migration of UnD-ESCs, but not that of D-ESCs, and found that enhanced MMP9 activity increased cellular migration in C-peptide-treated UnD-ESCs through β-catenin regulation. Under normal conditions in the presence of trophoblastic cells, elevated MMP9 expression enhanced cellular motility and invasive capacity in D-ESCs. Therefore, the decidua might contribute to the encapsulation of early conceptus and trophoblast invasion (Gellersen et al., 2010). The abnormally fast motility of UnD-ESCs might result in blockage of the D-ESC-embryo interface. Additionally, uncontrolled motility of UnD-ESCs can cause endometriosis, adenomyosis, and endometrial cancers (Mu et al., 2012; Zhang et al., 2016). Therefore, our data provide compelling evidence for the correlation between infertility and high C-peptide levels in patients with diabetes and PCOS (Joham et al., 2016; de Medeiros et al., 2018).

## Data Availability

The raw data supporting the conclusions of this article will be made available by the authors, without undue reservation.
