# Supplementary material for: C-Peptide Promotes Cell Migration by Controlling Matrix Metallopeptidase-9 Activity Through Direct Regulation of β-Catenin in Human Endometrial Stromal Cells
Source: Front Cell Dev Biol. 2022 Jan 21;10:800181. doi: 10.3389/fcell.2022.800181 (PMC8814361; doi:10.3389/fcell.2022.800181)
Supplement: Supplementary file 1 [file DataSheet1.docx]

Supplementary Material

**Supplementary Table 1. The human primers used in this study**

| Gene | Forward (5`-3`) | Reverse (5`-3`) |
| --- | --- | --- |
| GPR146 | GGACGCCACACTATCTGATCC | AGTGCAGTAGCCCCAGGTAG |
| MMP2 | TACAGGATCATTGGCTACACACC | GGTCACATCGCTCCAGACT |
| MMP9 | GGGACGCAGACATCGTCATC | TCGTCATCGTCGAAATGGGC |
| TIMP1 | CTTCTGCAATTCCGACCTCGCT | ACGCTGGTATAAGGTGGTCTG |
| TIMP2 | GCTGCGAGTGCAAGATCAC | TGGTGCCCGTTGATGTTCTTC |
| TIMP3 | CATGTGCAGTACATCCATACGG | CATCATAGACGCGACCTGTCA |
| GAPDH | GGAGCGAGATCCCTCCAAAAT | GGCTGTTGTCATACTTCTCATGG |
| Cyclo A | TGCCATCGCCAAGGAGTAG | TGCACAGACGGTCACTCAAA |

**Supplementary Table 2. ChIP wash Buffers used for ChIP-PCR assay**

| Wash Buffers | Buffer composition |
| --- | --- |
| I | 20 mM Tris-HCL, pH 8.0, 0.1% SDS, 1% Triton X-100, 2mM EDTA, 150 mM NaCl |
| II | Wash Buffer I with 500 mM NaCl |
| III | 10 mM Tris, pH 8.0, 0.25M LiCl, 1% Igepal CA-630 (NP-40) , 1% Na-Deoxycholate, 1 mM EDTA |
| IV (TE Buffer) | 10 mM Tris-HCL, pH 8.0, 1mM EDTA |

**Supplementary Table 3. Primers used for ChIP-PCR assay**

| Primer | Forward (5`-3`) | Reverse (5`-3`) |
| --- | --- | --- |
| MMP9-ChIP | TGTCCCCTTTACTGCCCTGA | GAAGACTCCCTGAGACTT |
| TIMP1-ChIP | CCCATATTTCCTCATCCGTA | CTTACCCAGGATCACAGAG |
| TIMP3-ChIP | CCCAGTCATGTCCAACCCA | CAGCTGGCTATGATATATG |

**Supplementary Figure 1. C-peptide enhances cell migration in undecidualized human endometrial stromal cells (UnD-ESCs) (related Fig. 1)**

The isolated HESCs were used as the undecidualized HESCs (UnD-ESCs) and decidualized HESCs (D-ESCs) on day 2 after the induction of decidualization in the presence of 8-Br-cAMP. UnD-ESCs or D-ESCs were serum-starved for 18 h, wounded using a T200 tip, and incubated with or without 1 nM C-peptide for 24 h. Then, HESCs were stained using the CytoPainter Cell Tracking Staining Kit and visualized using an Olympus CKX53 microscope (X4). Asterisks (*) indicate significant differences (P < 0.05) when compared to C-peptide untreated control or C-peptide treated UnD-ESCs control for 24 h. Scale bar = 100 μm.

**Supplementary Figure 2.** C-peptide did not affect GPR146 mRNA expression significantly **(related to Figure. 2)**

UnD-ESCs were incubated with or without 50 nM C-peptide for 24 h and subjected to qRT-PCR analysis for evaluating GPR146 and cyclo-A expression.

**Supplementary Figure 3. Confirmation of the effect of Akti on C-peptide-induced phosphorylation of Akt and β-catenin**

The undecidualized endometrial stromal cells (UnD-ESCs) were serum-starved for 48 hours, then pretreated with 1μΜ Akti, incubated with 50 nM C-peptide for 10 min, then lysed with cytoplasmic protein extraction buffer. Western blot analysis was performed on cell lysates.

**Supplementary Figure 4.** **Putative binding sites of β-catenin/TCF-1 in the promoter regions of MMP9, TIMP1, and TIMP3. (related to Figure. 4)**

**Supplementary Figure 5. β-catenin is required for C-peptide-induced migration in undecidualized human endometrial stromal cells (Fig. 5).**

(A) Undecidualized human endometrial stromal cells (UnD-ESCs) were transduced using shRNAs for scramble and β-catenin and selected for 5 days with 1.5 μM puromycin. The cells were lysed and subjected to western blot analysis. (B) UnD-ESCs were serum-starved for 18 h, wounded using a T200 tip, and then pretreated with 10 nM PNU-74654 for 1 h. The cells were incubated with or without 50 nM C-peptide for 24 h, visualized using the CytoPainter Cell Tracking Staining Kit and an Olympus CKX53 microscope (X4), and finally analyzed using ImageJ software. (C) UnD-ESCs were loaded onto trans-well filters and pretreated with 10 nM PNU-74654 for 1h. C-peptide (50 nM) was added to the outer chamber of each filter, and the cells were incubated for 24 h. The nucleus of the cells was stained with DAPI and the number of cells was counted using Image J. Asterisks (*) indicate significant differences (P < 0.05) when compared to the C-peptide-untreated UnD-ESCs control or C-peptide-treated UnD-ESCs control. Scale bar = 100 μm. shScram, shRNA for Scramble; shCTNNB1, shRNA for β-catenin.

**Supplementary Figure 6. Akt and PP1 are important for C-peptide-induced migration in undecidualized human endometrial stromal cells (related to Fig. 5)**

(A) The undecidualized human endometrial stromal cells (UnD-ESCs) were serum-starved for 18 h, wounded using a T200 tip, pretreated with 1 μΜ Akti or 10 nM OA, and then incubated (24 h) with or without 50 nM C-peptide. The cells were visualized using the CytoPainter Cell Tracking Staining Kit and an Olympus CKX53 microscope (X4) and analyzed using ImageJ. (B) UnD-ESCs were transduced using shRNAs for scramble and PPP1C and selected for 5 days with 1.5 μM puromycin. The cells were serum-starved for 18 h, wounded using a T200 tip, and incubated with or without 50 nM C-peptide for 24 h. The cells were visualized using the CytoPainter Cell Tracking Staining Kit and an Olympus CKX53 microscope (X4) and then analyzed using ImageJ. (C) UnD-ESCs were loaded onto trans-well filters and pretreated with 1 μΜ Akti or 10 nM OA for 1h. C-peptide was added to the outer chamber of each filter and the cells were incubated for 24 h. The nucleus of the cells was stained with DAPI. Asterisks (*) indicate significant differences (P < 0.05) when compared to C-peptide untreated UnD-ESCs control or C-peptide treated UnD-ESCs control. Scale bar = 100 μm. shScram, shRNA for Scramble; shPPP1C, shRNA for catalytic subunit alpha isozyme of protein phosphatase 1.
